# Development of a reverse transcription loop-mediated isothermal amplification based clustered regularly interspaced short palindromic repeats Cas12a assay for duck Tembusu virus

**DOI:** 10.3389/fmicb.2023.1301653

**Published:** 2023-11-30

**Authors:** Yangbao Ding, Zhanhong Huang, Xinbo Li, Mei Tang, Weiqiang Li, Siyu Feng, Luxiang Zhao, Junsheng Zhang, Shichao Yuan, Fen Shan, Peirong Jiao

**Affiliations:** ^1^College of Veterinary Medicine, Guangdong Laboratory for Lingnan Modern Agriculture, South China Agricultural University, Guangzhou, China; ^2^Key Laboratory of Animal Vaccine Development, Ministry of Agriculture and Rural Affairs, Guangzhou, China; ^3^Guangdong Provincial Key Laboratory of Zoonosis Prevention and Control, Guangzhou, China; ^4^Guangzhou Collaborative Innovation Center on Science-Tech of Ecology and Landscape, Guangzhou Zoo, Guangzhou, China

**Keywords:** duck, Tembusu virus, RT-LAMP, CRISPR-Cas12a, clinical field detection

## Abstract

Duck Tembusu virus (DTMUV) is an emerging pathogen that poses a serious threat to the duck industry in China. Currently, polymerase chain reaction (PCR), quantitative PCR (qPCR) and reverse transcription loop-mediated isothermal amplification (RT-LAMP) are commonly used for DTMUV detection. However, these methods require complex steps and special equipment and easily cause false-positive results. Therefore, we urgently need to establish a simple, sensitive and specific method for the clinical field detection of DTMUV. In this study, we developed an RT-LAMP-based CRISPR-Cas12a assay targeting the *C* gene to detect DTMUV with a limited detection of 3 copies/μL. This assay was specific for DTMUV without cross-reaction with other common avian viruses and only required some simple pieces of equipment, such as a thermostat water bath and blue/UV light transilluminator. Furthermore, this assay showed 100% positive predictive agreement (PPA) and negative predictive agreement (NPA) relative to SYBR Green qPCR for DTMUV detection in 32 cloacal swabs and 22 tissue samples, supporting its application for clinical field detection.

## 1 Introduction

Tembusu virus (TMUV) is a single-stranded, positive-sense RNA virus, that belongs to the genus *Flavivirus* in the family *Flaviviridae* (Zhang et al., 2017). The genome of TMUV is formed by a 5′-terminal noncoding region (NCR), a single open reading frame (ORF) encoding polyprotein and a 3′-terminal noncoding region (NCR). The polyprotein is cleaved into three structural proteins (capsid *C*, membrane precursor *prM*, and envelope *E*) and seven nonstructural proteins (*NS1*, *NS2A*, *NS2B*, *NS3*, *NS4A*, *NS4B*, and *NS5*) by viral and host proteases (Hamel et al., 2021; Xu et al., 2023). The virus was first isolated from Culex tritaeniorhynchus mosquitoes in Malaysia in 1955 and then infected a wide range of birds, including ducks, chickens, geese, pigeons and sparrows, due to its cross-species transmission (Yu et al., 2018). Among these hosts, ducks are the most susceptible animals to TMUV infection, and duck-origin TMUV is abbreviated as DTMUV (Tang et al., 2012; Zhu et al., 2012; Thontiravong et al., 2015; Yan et al., 2017; Tunterak et al., 2018). Domestic ducks infected with DTMUV exhibit diarrhea, anorexia, encephalitis, paralysis, and a significant decline in egg production, resulting in a mortality rate ranging from 5% to 30% (Yan P. X. et al., 2011; Liang et al., 2016; Li et al., 2020). Since the first report of DTMUV in China in 2010, it has infected more than 130 million domestic ducks and presents a grave danger to the duck industry (Lei et al., 2017). Herein, the diagnosis and prevention of DTMUV infection are essential for the duck industry. In addition to clinical diagnosis methods, the molecular diagnosis methods, including PCR-based assays, qPCR-based assays and isothermal amplification-based assays are commonly used for DTMUV infection. Currently, several PCR-based assays, targeting the *E*, *NS3* and *NS5* genes have been established for DTMUV detection (Zang et al., 2012; Li, 2013; Wang et al., 2017). In addition, some qPCR-based assays targeting the *E*, *NS1* and 3′-NCR genes were also exploited to detect DTMUV (Yan et al., 2012; Yun et al., 2012; Chen G. Q. et al., 2018; Li et al., 2023). However, these PCR-based and qPCR-based assays are mainly applied to laboratory testing of DTMUV. To develop clinical field detection methods, several RT-LAMP assays targeting the *E* or *NS5* gene were established for DTMUV detection (Jiang et al., 2012; Wu et al., 2017; Wang et al., 2022). The assays effectively amplify target genes with four to six primers within 1 h, and their products are visualized by the naked eye via turbidity or dye indicators (Yan et al., 2020; Hongjaisee et al., 2021; Soroka et al., 2021). However, these assays easily cause false-positive results owing to their nonspecific amplification (Soroka et al., 2021). Therefore, we urgently need to establish a sensitive and specific molecular diagnostic method for the clinical field detection of DTMUV.

Recently, the Cas12a (Cpf1), a member of the clustered regularly interspaced short palindromic repeats (CRISPR)-associated system (Cas), was used for the molecular diagnosis of pathogenic microorganisms (Chertow, 2018; Li et al., 2018b; Liang et al., 2019). The Cas12a recognizes the target DNA under the guidance of CRISPR-derived RNA (crRNA) complementary to the target DNA sequence with T nucleotide-rich PAM (5′-TTTV-3′) and some suboptimal PAMs (5′-TCTV-3′, 5′-TTV-3′, 5′-CCCV-3′ and 5′-TCCV-3′) (Dronina et al., 2022). Subsequently, the Cas12a performs the collateral cleavage on nonspecific single-stranded DNA (ssDNA) (e.g., the fluorescent ssDNA reporter HEX-N12-BHQ1) when the Cas12a, crRNA, and target DNA form a ternary complex (Gootenberg et al., 2018; Li et al., 2018b). Based on this collateral cleavage activity, a number of Cas12a-based molecular diagnostic methods coupled with isothermal amplification techniques containing RPA, RCA, RAA and LAMP have been developed to detect pathogens, including RNA viruses, DNA viruses and bacteria (Li et al., 2018b; Xu et al., 2020; Mayuramart et al., 2021; Chen et al., 2022; Qin et al., 2022; de Sousa et al., 2023; Fan et al., 2023; Mao et al., 2023; Wu et al., 2023; Zhao et al., 2023). For instance, an RT-RAA-based CRISPR-Cas12a assay was established to detect SARS-CoV-2 and influenza viruses, and the assay was effective for screening of these viruses (Mayuramart et al., 2021). Another RT-RAA-based CRISPR-Cas12a assay was also developed to detect SARS-CoV-2 on automated centrifugal microfluidics, and this assay achieved ultrasensitive SARS-CoV-2 detection using two crRNAs to simultaneously match two sites of the target DNA (Chen et al., 2022). In addition, a LAMP-based CRISPR-Cas12a assay was exploited to detect African swine fever virus (ASFV), and this assay performed high sensitivity and specificity for the diagnosis of ASFV (Qin et al., 2022). Furthermore, an RCA-based CRISPR-Cas12a assay was exploited for methicillin-resistant *Staphylococcus aureus* detection, and this assay was applied to point-of-care testing (Xu et al., 2020). Altogether, these assay-based CRISPR-Cas12a methods have higher specificity and sensitivity than conventional PCR-based assays, qPCR-based assays and isothermal amplification-based assays, and are suitable for clinical field detection.

In this study, we established an RT-LAMP-based CRISPR-Cas12a assay to detect DTMUV infection. In addition, we evaluated its performance in terms of specificity and sensitivity, and validated its clinical field detection with cloacal swabs and tissue samples taken from DTMUV-infected ducks.

## 2 Materials and methods

### 2.1 Viruses and clinical samples

Duck Tembusu virus (DTMUV, S132), H5N6 highly pathogenic avian influenza virus (H5N6 AIV, S230), H7N9 highly pathogenic avian influenza virus (H7N9 AIV, Q26), H9N2 avian influenza virus (H9N2 AIV, S448), infectious bronchitis virus (IBV, M41), and Newcastle disease virus (NDV, S391) were isolated by our laboratory. The 54 clinical samples, including 32 cloacal swabs and 22 tissue samples, were randomly taken from the ducklings infected with DTMUV in the animal infection experiment.

### 2.2 RNA extraction and cDNA synthesis

Total RNA was extracted from these viruses and clinical samples using a Fast Total RNA Extraction Kit (Fastagen). Then, these total RNAs were utilized to synthesize cDNAs with PrimeScript^™^ II 1st Strand cDNA Synthesis Kit (Takara). Finally, the obtained RNAs and cDNAs were preserved at a temperature of −80°C for further experimental investigation.

### 2.3 SYBR Green qPCR assay

The SYBR Green qPCR assay previously established by our laboratory was used for DTMUV detection in the 54 clinical samples mentioned above. Each 20 μL SYBR Green qPCR consisted of 10 μL 2× GoTaq^®^ qPCR Master Mix (Promega), 10 μM NS5-F (forward primer), 10 μM NS5-R (reverse primer) and 5 μL cDNA (Table 1). Then, the reaction was conducted on a Bio-Rad CFX96 system (Bio-Rad) using the following thermal profile: 95°C for 2 min and 40 cycles of 95°C for 15 s and 58°C for 30 s. A sample with an average Ct value ≤35 was positive, and a sample with an average Ct value >35 or undetectable DTMUV RNA level was negative.

**Table 1:** Primers and crRNAs used in this study.

Primers	Sequences (5′-3′)
F3	AAAAACCAGGAAGACCCGG
B3	CCAACCAGCTTCCATCTCTT
FIP	TCAGGACCCCATCAATCGTCCTATATGCTAAAGCGCGGAACG
BIP	ACCCATAAGGTTTGTGCTGGCTTTCCAATGGTTGGCCTCAG
Loop-F	ATCCGCGCTAGCGGATTT
Loop-B	ACTGACTTTYTTCAAGTTYACAGCC
NS5-F	CTAGGGGCGTTGAAGAGTTCTG
NS5-R	ATGTGTTGAATTACGCGAAAGTGG
crRNA-1	GGGUAAUUUCUACUAAGUGUAGAUUGCUGGCUCUACUGACUUUC
crRNA-2	GGGUAAUUUCUACUAAGUGUAGAUUGGGUCCUGCUCCUCUCAGG
crRNA-3	GGGUAAUUUCUACUAAGUGUAGAUAUGGGGUCCUGAGAGGAGCA

In addition, the limited detection of this SYBR Green qPCR assay was determined. Briefly, the copy numbers of standard plasmid DNA containing the *C* gene of DTMUV previously constructed by our laboratory were calculated via the NEBioCalculator with the formula: DNA copy numbers = (*M* × 6.022 × 10^23^)/(*N* × 10^9^ × 660), in which *M* represented the amount of DNA in nanograms, *N* was the number of base pairs in a single standard plasmid DNA. Then, this DNA was diluted in a tenfold series (10^5^ to 10^0^ copies/μL) based on its copy numbers. Finally, 1 μL of tenfold serial dilution was subjected to the SYBR Green qPCR assay with three repeats.

### 2.4 Establishment and optimization of the RT-LAMP assay

Thirty whole genome sequences of DTMUV isolated in China were downloaded from GenBank and aligned by MegAlign (LaserGene 7.0 software, DNAStar) to find conserved regions. Based on the conserved region, the RT-LAMP primers, including FIP, BIP, F3, B3, Loop-F and Loop-B, were designed by PrimerExplorer V5 and synthesized by GENEWIZ. According to the recommendation of the NEB company, we initially established an RT-LAMP reaction containing 2.5 μL 10× isothermal amplification buffer (NEB), 1.6 μM each of FIP and BIP primers, 0.2 μM each of F3 and B3 primers, 0.4 μM each of Loop-F and Loop-B primers, 6 mM MgSO_4_ (NEB), 1.4 mM dNTP (Promega), 7.5 units WarmStart^®^ RTx Reverse Transcriptase (NEB), 8 units Bst 2.0 WarmStart^®^ DNA Polymerase (NEB) and 2 μL RNA. The total volume of this reaction was adjusted to 25 μL with sterile deionized water. Then, the reaction was performed at 65°C for 50 min.

The RT-LAMP assay was optimized via a variable-controlling approach with different concentrations of loop primers, MgSO_4_ and dNTPs, different ratios of outer primers (F3 and B3 primers) to inner primers (FIP and BIP primers), different reaction times, and different reaction temperatures. Two microlitre of RT-LAMP product was utilized for 1% agarose gel electrophoresis, and the grayscale value of each agarose gel electrophoretic band was measured by ImageJ software (National Institutes of Health).

### 2.5 Evaluation of specificity and sensitivity for the RT-LAMP assay

To evaluate the specificity of this RT-LAMP assay, H5N6 AIV, H7N9 AIV, H9N2 AIV, IBV, and NDV were utilized to determine whether these viruses were able to be falsely detected. Briefly, 1 μL RNA of each virus was subjected to this assay with three repeats, and 2 μL of RT-LAMP product was utilized for 1% agarose gel electrophoresis.

To evaluate the sensitivity of this assay, the copy numbers of DTMUV RNA were firstly calculated by NEBioCalculator with the formula: RNA copy numbers = (*M* × 6.022 × 10^23^)/(*N* × 10^9^ × 340), in which *M* represented the amount of RNA in nanograms, *N* was the length of the RNA in base. Then, the DTMUV RNA was diluted to 10^9^, 10^8^, 10^7^, 10^6^, 10^5^, 10^4^, 10^3^, 10^2^, 10, 9, 8, 7, 6, 5, 4, 3 and 2 copies/μL, and 1 copy/μL based on its copy numbers. Finally, 1 μL of tenfold serial dilution was subjected to this RT-LAMP assay with three repeats, and 2 μL of RT-LAMP product was utilized for 1% agarose gel electrophoresis.

### 2.6 Preparation of Cas12a crRNAs

CRISPR-derived RNAs (crRNAs) complementary to the target sequences located on the *C* gene of DTMUV with T nucleotide-rich PAM (5′-TTTV-3′) and suboptimal PAM (5′-TTV-3′) were designed by CRISPR-DT software. The crRNAs were chemically synthesized by GenScript (Nanjing, China).

### 2.7 CRISPR-Cas12a-mediated cleavage assay

The 20 μL of CRISPR-Cas12a-mediated cleavage reaction contained 100 nM Cas12a (NEB), 125 nM crRNA, 500 nM HEX-N12-BHQ1 (HuicH), 40 units RNasin^®^ Ribonuclease Inhibitors (Promega), 2 μL NEBuffer^™^ r2.1 (NEB), and 2 μL RT-LAMP product. The reaction was performed at 37°C for 30 min, and the result was obtained via a blue/UV light transilluminator (Thermo Fisher). In addition, the product of this assay was transferred into a 96-well plate containing 80 μL sterile-deionized water and then monitored for fluorescence intensity by a microplate reader (Promega) at wavelengths from 533 to 559 nm.

### 2.8 Optimization of the cleavage time of CRISPR-Cas12a

To optimize the cleavage time of CRISPR-Cas12a, the cleavage reactions were performed at 37°C, and fluorescence results were read at 5, 10, 15, 20, 25 and 30 min, respectively. The reactions corresponding to each cleavage time were conducted with three repeats. The results were obtained using a blue/UV light transilluminator. In addition, the products of these reactions were transferred into a 96-well plate containing 80 μL sterile-deionized water and then monitored for fluorescence intensity by a microplate reader at wavelengths from 533 to 559 nm.

### 2.9 Evaluation of specificity and sensitivity for the RT-LAMP-based CRISPR-Cas12a assay

To evaluate the specificity of the RT-LAMP-based CRISPR-Cas12a assay, H5N6 AIV, H7N9 AIV, H9N2 AIV, IBV and NDV were used to determine whether these viruses could be falsely detected. Briefly, 1 μL RNA of each virus was subjected to this assay with three repeats. The results were obtained using a blue/UV light transilluminator.

To evaluate the sensitivity of this RT-LAMP-based CRISPR-Cas12a assay, the copy number of DTMUV RNA was calculated by the same formula mentioned above. Then, the DTMUV RNA was diluted to 10^5^, 10^4^, 10^3^, 10^2^, 10, 9, 8, 7, 6, 5, 4, 3 and 2 copies/μL, and 1 copy/μL based on its copy numbers. Finally, 1 μL of tenfold serial dilution was subjected to this assay with three repeats. The results were obtained using the blue/UV light transilluminator.

### 2.10 RT-LAMP-based CRISPR-Cas12a assay for clinical sample detection

The 54 clinical samples taken from DTMUV-infected ducklings, including 22 cloacal swabs and 32 tissue samples, were used to detect DTMUV by RT-LAMP-based CRISPR-Cas12a assay. This assay was run in parallel with the SYBR Green qPCR assay for these clinical samples. The results were obtained with the blue/UV light transilluminator. Then, the results were compared with those obtained with the SYBR Green qPCR assay as mentioned above.

### 2.11 Statistical analysis

All data were analyzed with GraphPad Prism 5.0 (La Jolla, CA, United States). Significant differences were evaluated using a two-way analysis of variance (ANOVA). *p*-values <0.05 were considered statistically significant. Statistical significance is indicated as follows: ^*^p < 0.05, ^**^p < 0.01, and ^***^p < 0.001, and no significant difference is represented as “ns.”

### 2.12 Ethics statement

All animal experiments were approved via the Laboratory Animal Ethics Committee of South China Agricultural University. Highly pathogenic avian influenza viruses were conducted in the BSL-3 facility.

## 3 Results

### 3.1 Workflow of the RT-LAMP-based CRISPR-Cas12a assay

**Figure 1:**
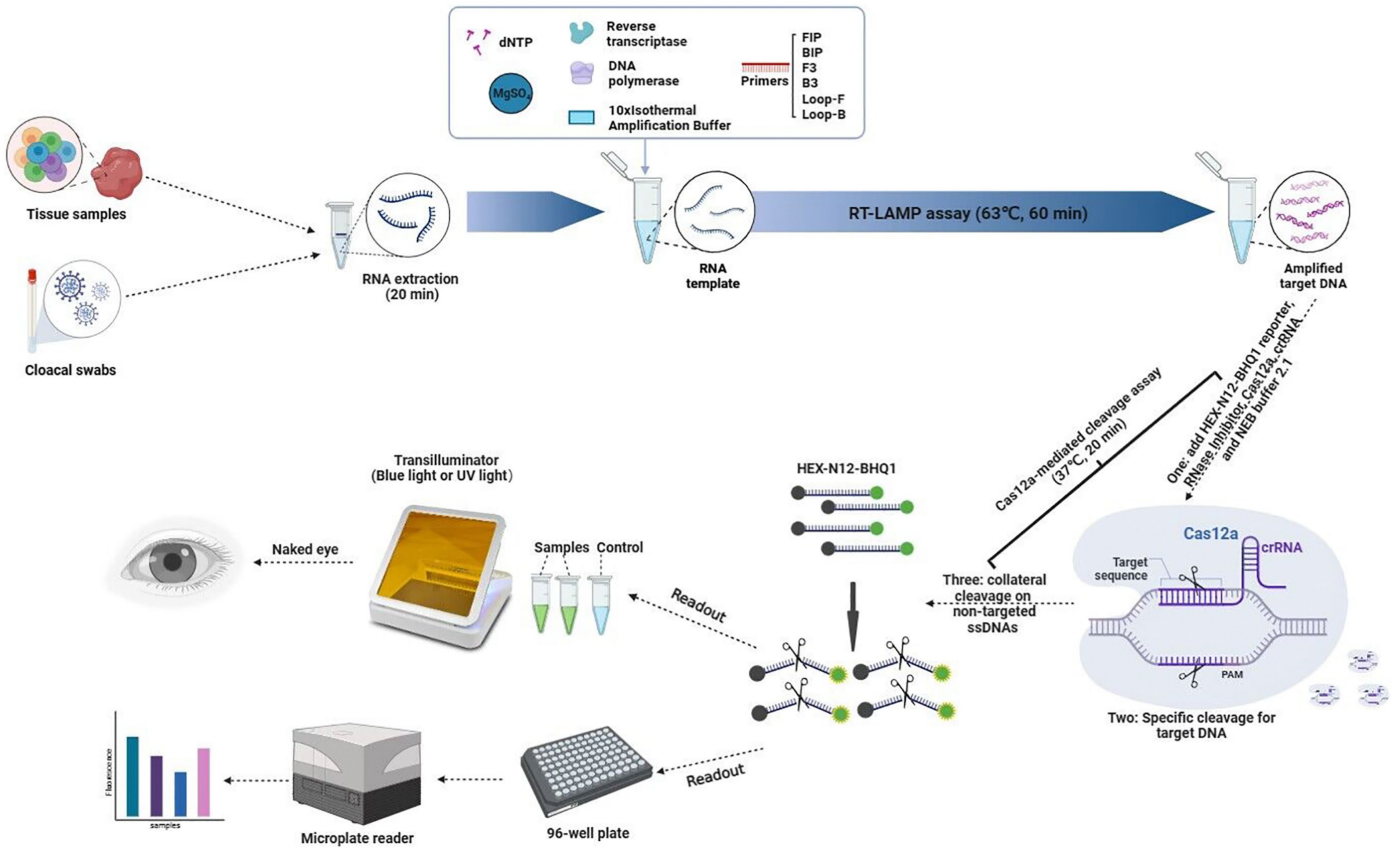
Schematic illustration of the workflow for the RT-LAMP-based CRISPR-Cas12a assay.

In this study, cloacal swabs or tissue samples were used for rapid extraction of RNA within 20 min. Then, the target DNA was amplified at 63°C for 60 min by the RT-LAMP assay using the extracted RNA and cleaved at 37°C for 20 min by CRISPR Cas12a under the guidance of specific crRNA. Under this condition, the collateral cleavage activity of Cas12a for ssDNA without selectivity was activated, and then the fluorescent ssDNA reporter HEX-N12-BHQ1 was cleaved by it. The resulting fluorescence was detected by a simple blue light transilluminator, UV light transilluminator or microplate reader (Figure 1).

### 3.2 Establishment of the RT-LAMP assay

**Figure 2:**
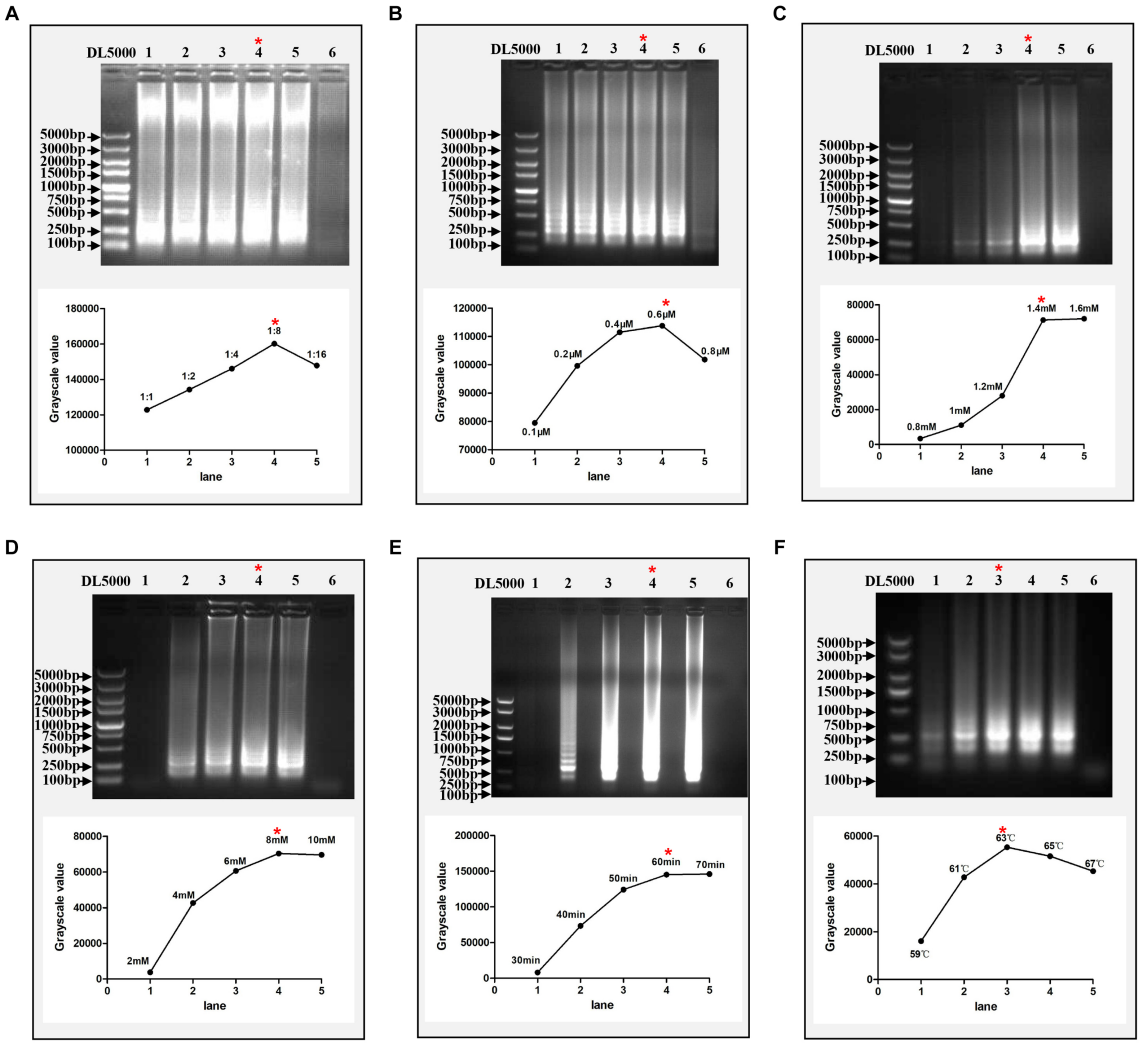
Optimization of the RT-LAMP assay. **(A)** Optimization of the ratio of outer primers to inner primers. Lanes 1–5 represent 1:1, 1:2, 1:4, 1:8, and 1:16, respectively. **(B)** Optimization of the concentration of loop primers. Lanes 1–5 represent 0.1, 0.2, 0.4, 0.6, and 0.8 μM, respectively. **(C)** Optimization of the concentration of dNTPs. Lanes 1–5 represent 0.8, 1, 1.2, 1.4, and 1.6 mM, respectively. **(D)** Optimization of the concentration of MgSO_4_. Lanes 1–5 represent 2, 4, 6, 8, and 10 mM, respectively. **(E)** Optimization of reaction time. Lanes 1–5 represent 30, 40, 50, 60, and 70 min, respectively. **(F)** Optimization of reaction temperature. Lanes 1–5 represent 59, 61, 63, 65, and 67°C, respectively. In each figure, Lane 6 represents the negative control and the red asterisk indicates the optimal conditions for this assay. Each reaction was performed with three repeats, and one representative readout is shown in the figures. The grayscale value of each agarose gel electrophoretic band was measured by ImageJ software (National Institutes of Health).

Thirty whole genome sequences of DTMUV isolated in China were downloaded from NCBI GenBank and aligned to find their conservative regions. As a result, the nucleotide sequences of the *C* genes for these DTMUV were relatively conserved (Supplementary Figure S1B). Based on their conserved regions, a set of specific RT-LAMP primers, including F3, B3, FIP, BIP, Loop-F and Loop-B, were designed (Supplementary Figures S1A,B; Table 1). The RT-LAMP reaction system recommended by the NEB company was used as the standard for optimizing each component in this system. The results showed that the optimal concentrations of loop primers, dNTPs and MgSO_4_ were 0.6 μM, 1.4 mM and 8 mM, respectively (Figures 2B–D). In addition, the optimal ratio of outer primers to inner primers was 1:8 in this RT-LAMP reaction system (Figure 2A). Meanwhile, the optimal reaction time and reaction temperature for this system are 60 min and 63°C, respectively (Figures 2E,F).

### 3.3 Specificity and sensitivity of the RT-LAMP assay

**Figure 3:**
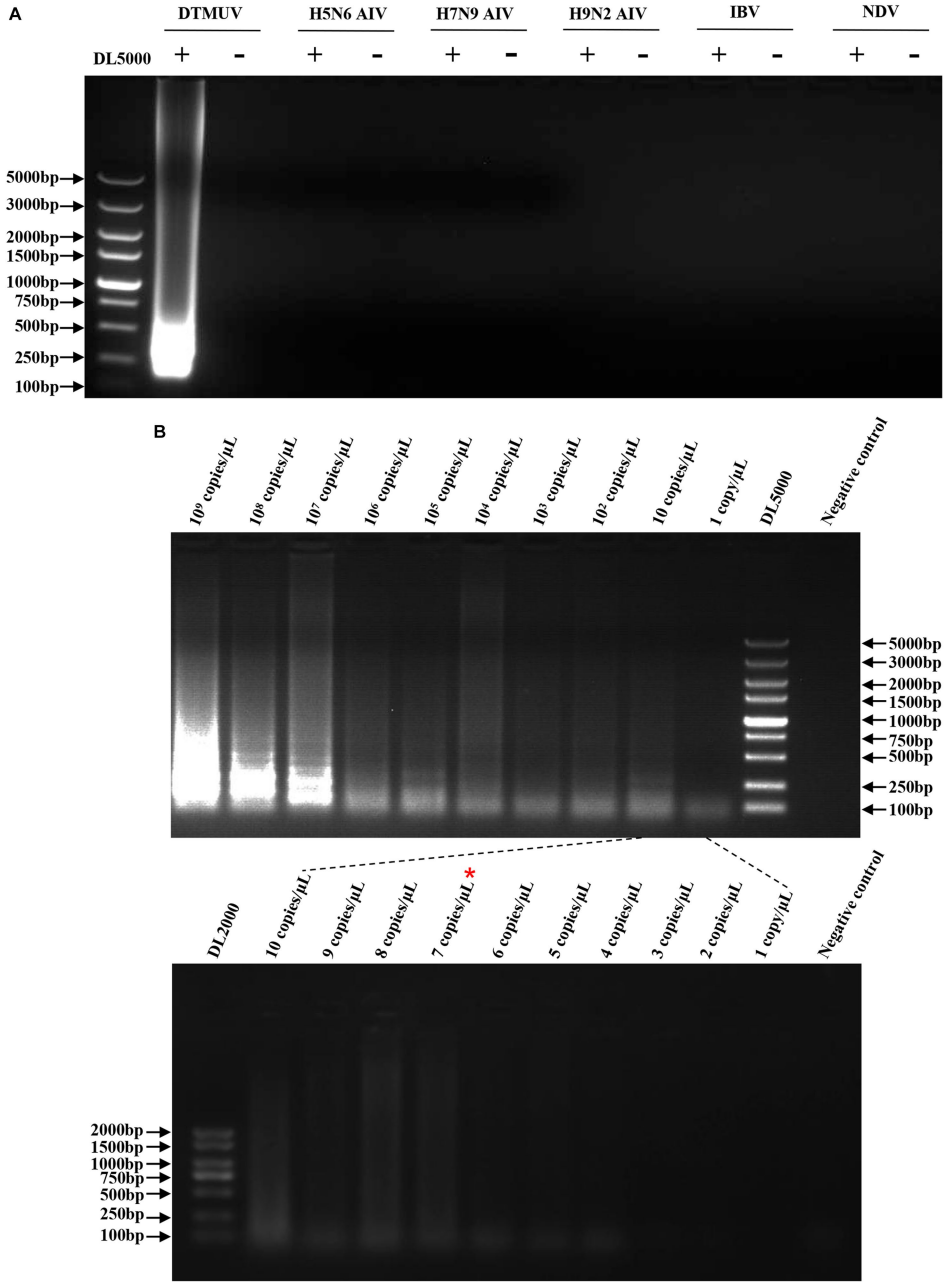
Specificity and sensitivity of the RT-LAMP assay. **(A)** Specificity of the RT-LAMP assay. The plus sign (+) and minus sign (−) represent positive samples and negative controls, respectively. Each reaction was performed with three repeats, and one representative readout is shown in the figure. **(B)** Sensitivity of the RT-LAMP assay. Serial dilutions of DTMUV RNA from 10^9^ copies/μL to 1 copy/μL were used in this assay. Each dilution was performed with three repeats, and one representative readout is shown in the figure.

To evaluate the specificity of the RT-LAMP assay, the RNAs of H5N6 AIV, H7N9 AIV, H9N2 AIV, IBV, and NDV were used for this assay with specific primers of DTMUV. The results showed that the RT-LAMP reaction with DTMUV RNA generated the expected product, and no product was observed for the RT-LAMP reaction with other viral RNAs, which indicated that this RT-LAMP assay was specific for DTMUV (Figure 3A). To evaluate the sensitivity of this assay, the DTMUV RNA was serially diluted from 10^9^ copies/μL to 1 copy/μL. We found that its limit of detection was 7 copies/μL (Figure 3B).

### 3.4 CRISPR-Cas12a-mediated cleavage assay

**Figure 4:**
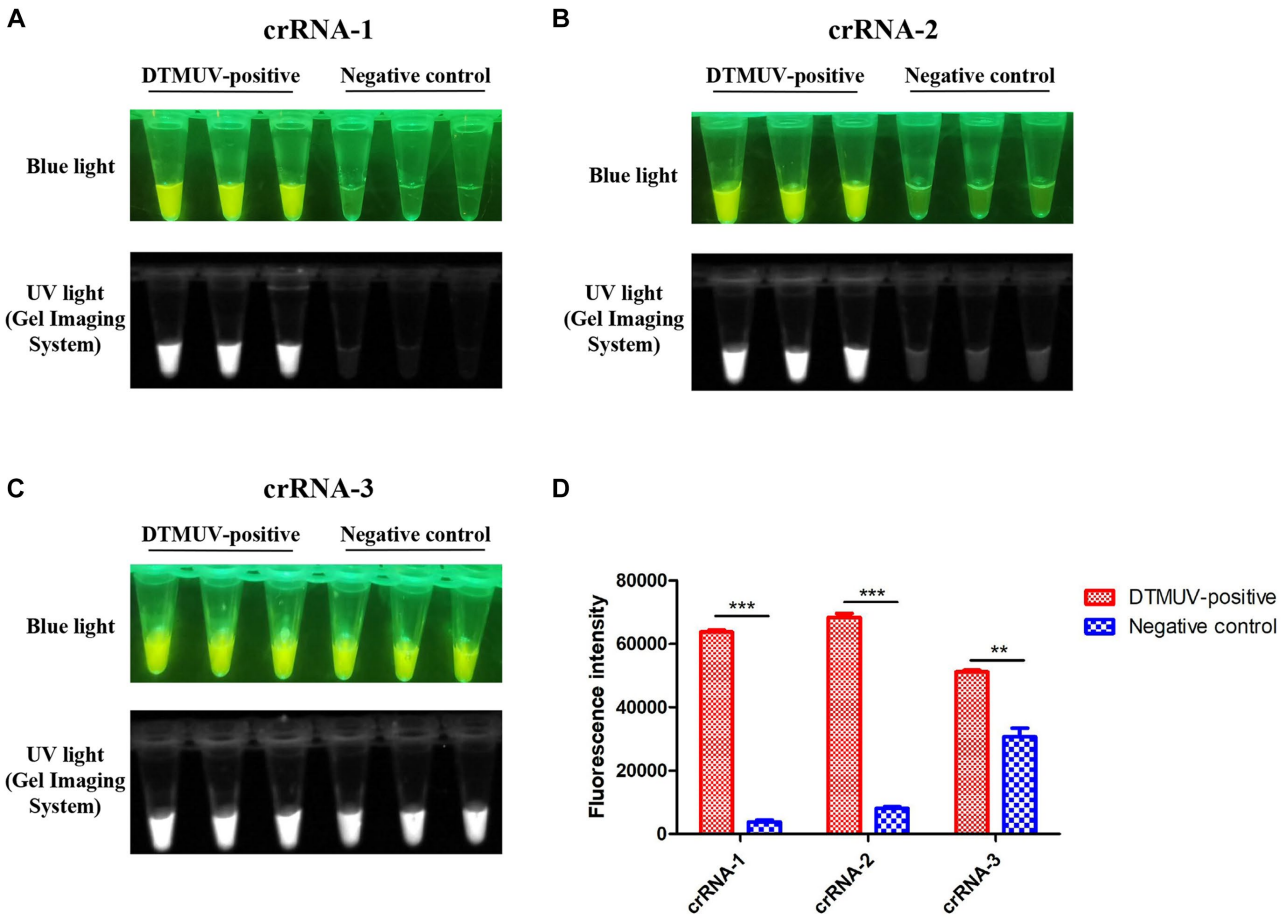
CRISPR-Cas12a-mediated cleavage assay. **(A–C)** crRNA-1, crRNA-2 and crRNA-3 targeting the *C* gene of DTMUV were used for the CRISPR-Cas12a-mediated cleavage assay. Each crRNA was performed with three repeats in this assay. **(D)** The fluorescence intensity value of each reaction was measured by a microplate reader. All data were analyzed with GraphPad Prism 5.0 (La Jolla, CA, United States). Significant differences were evaluated using a two-way analysis of variance (ANOVA). A *p*-value <0.05 was considered statistically significant. Statistical significance is indicated in the figure as follows: ^**^p < 0.01 and ^***^p < 0.001.

In this study, three crRNAs, crRNA-1, crRNA-2 and crRNA-3, located on the target DNA were designed and tested for the Cas12a-mediated cleavage assay (Table 1). Among them, crRNA-1 performed better than crRNA-2 and crRNA-3 because of its lower fluorescence background for the negative control in this assay (Figures 4A–D). crRNA-3 was not specific enough for the target DNA due to its high fluorescence background for the negative control (Figures 4C,D).

### 3.5 Optimization of the cleavage time of the CRISPR-Cas12a-mediated assay

**Figure 5:**
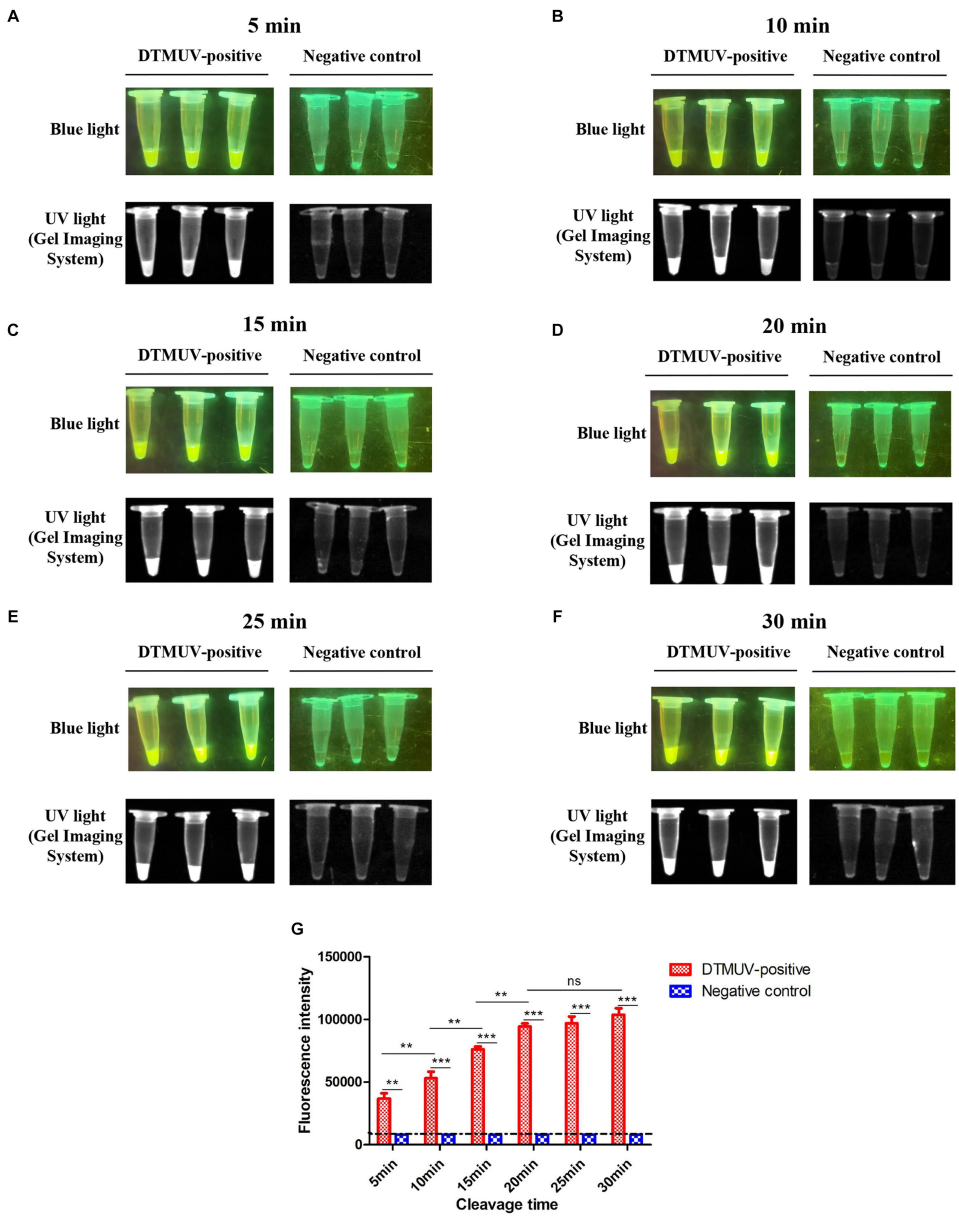
Optimization for cleavage time of the CRISPR-Cas12a-mediated assay. **(A–F)** Cleavage results of the CRISPR-Cas12a-mediated assay at different times. The reaction was performed with three repeats at each cleavage time. **(G)** The fluorescence intensity value of each reaction was measured by a microplate reader. All data were analyzed with GraphPad Prism 5.0 (La Jolla, CA, United States). Significant differences were evaluated using a two-way analysis of variance (ANOVA). A *p*-value <0.05 was considered to indicate a significant difference. Statistical significance is indicated in the figure as follows: ^**^p < 0.01, ^***^p < 0.001, and no significant difference is represented as “ns.”

To find the optimal cleavage time of this assay, the fluorescence intensity values corresponding to different time points were measured. We found that the average fluorescence intensity values at 5, 10, 15 and 20 min were 10^4.57^, 10^4.72^, 10^4.88^ and 10^4.98^, respectively, which indicated that the cleavage efficiency of CRISPR-Cas12a significantly increased with the extension of cleavage time within 20 min (*p* < 0.01) (Figures 5A–D,G). However, the average fluorescence intensities at 20, 25 and 30 min showed no significant change (Figures 5D–G). In summary, the best cleavage time of this assay was 20 min.

### 3.6 Specificity and sensitivity of the RT-LAMP-based CRISPR-Cas12a assay

**Figure 6:**
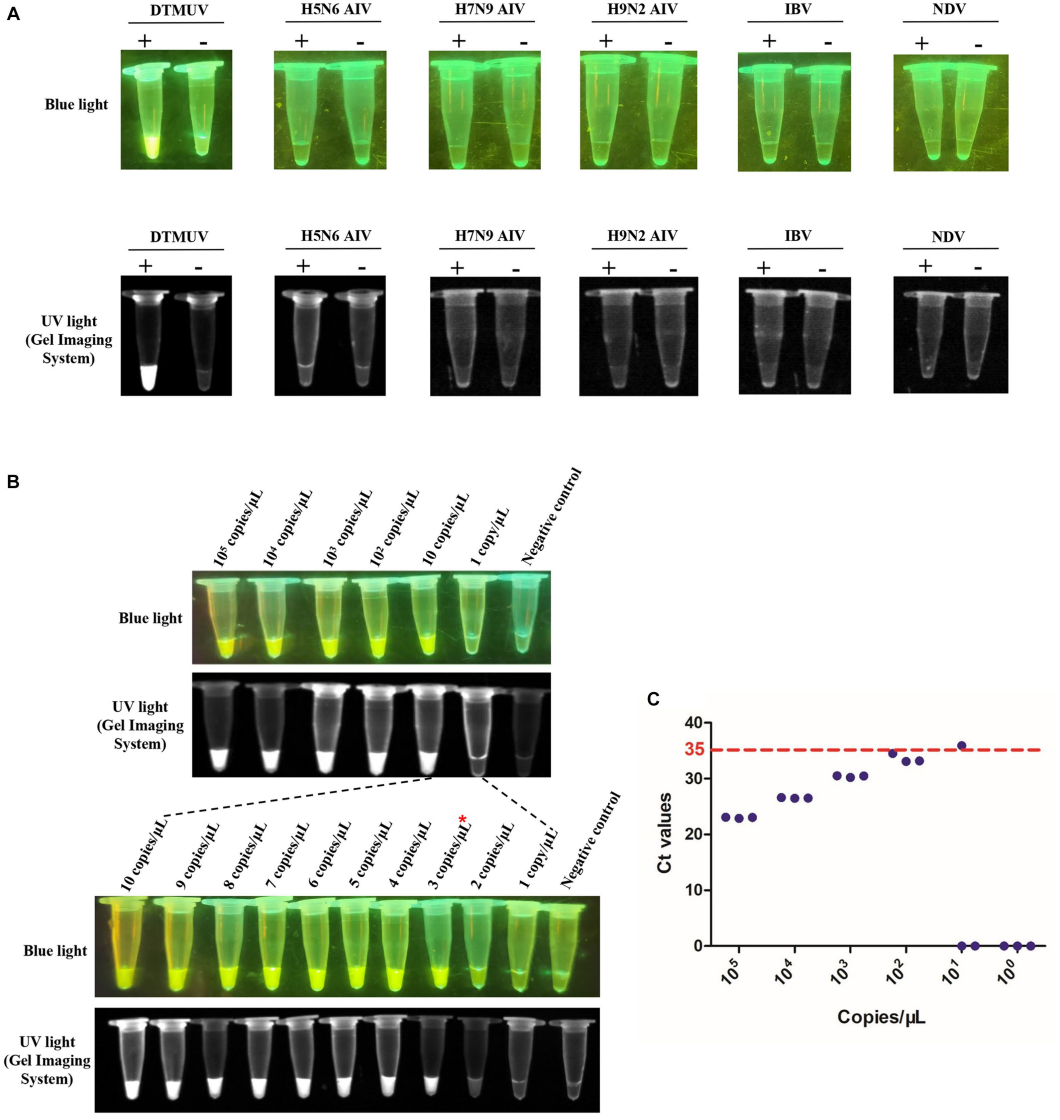
Specificity and sensitivity of the RT-LAMP-based CRISPR-Cas12a assay. **(A)** Specificity of the RT-LAMP-based CRISPR-Cas12a assay. The plus sign (+) and minus sign (−) represent positive samples and negative controls, respectively. Each reaction was performed with three repeats, and one representative readout is shown in the figure. **(B)** Sensitivity of the RT-LAMP-based CRISPR-Cas12a assay. Serial dilutions of DTMUV RNA from 10^5^ copies/μL to 1 copy/μL were used in this assay. Each reaction was performed with three repeats, and one representative readout is shown in the figure. **(C)** Sensitivity of SYBR Green qPCR. Tenfold serial dilutions of DTMUV RNA from 10^5^ copies/μL to 1 copy/μL were used in this assay. Each reaction was performed with three repeats. A sample with an average Ct value ≤35 was positive, and a sample with an average Ct value >35 or undetectable DTMUV RNA level was negative.

To evaluate the specificity of the RT-LAMP-based CRISPR-Cas12a assay, the RNAs of H5N6 AIV, H7N9 AIV, H9N2 AIV, IBV and NDV were used for this assay with specific primers and crRNA of DTMUV. We found that only the CRISPR-Cas12a reaction with the RT-LAMP product corresponding to DTMUV RNA emitted fluorescence, which indicated that this RT-LAMP-based CRISPR-Cas12a assay was specific for DTMUV (Figure 6A). To evaluate the sensitivity of this assay, the DTMUV RNA was serially diluted from 10^5^ copies/μL to 1 copy/μL. We found that its limited detection reached 3 copies/μL for DTMUV RNA (Figure 6B). Furthermore, this assay exhibited higher sensitivity compared to SYBR Green qPCR, whose limited detection was 100 copies/μL for standard plasmid DNA of DTMUV (Figure 6C).

### 3.7 Evaluation of the RT-LAMP-based CRISPR-Cas12a assay on clinical samples

**Figure 7:**
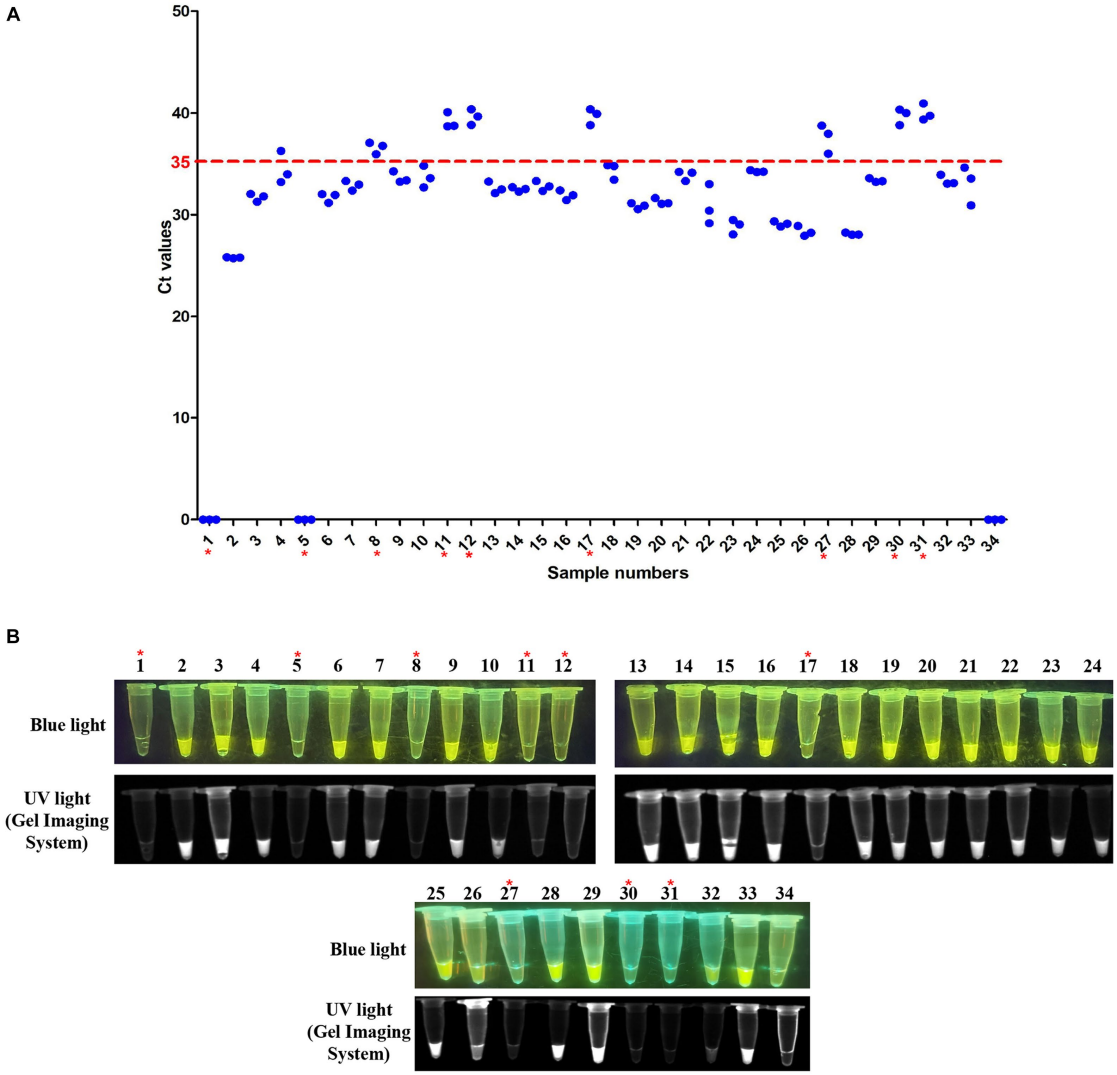
Evaluation of the RT-LAMP-based CRISPR-Cas12a assay with cloacal swabs. **(A)** SYBR Green qPCR assay on cloacal swabs. Numbers 1–32 represent cloacal swab identification numbers. Numbers 33 and 34 represent the positive sample and negative control, respectively. A sample with an average Ct value ≤35 was positive, and a sample with an average Ct value >35 or undetectable DTMUV RNA level was negative. **(B)** RT-LAMP-based CRISPR-Cas12a assay on cloacal swabs. Lanes 1–32 represent cloacal swabs. Lanes 33 and 34 represent the positive sample and negative control, respectively. In each figure, the red asterisk indicates the negative samples identified by the two assays.

**Figure 8:**
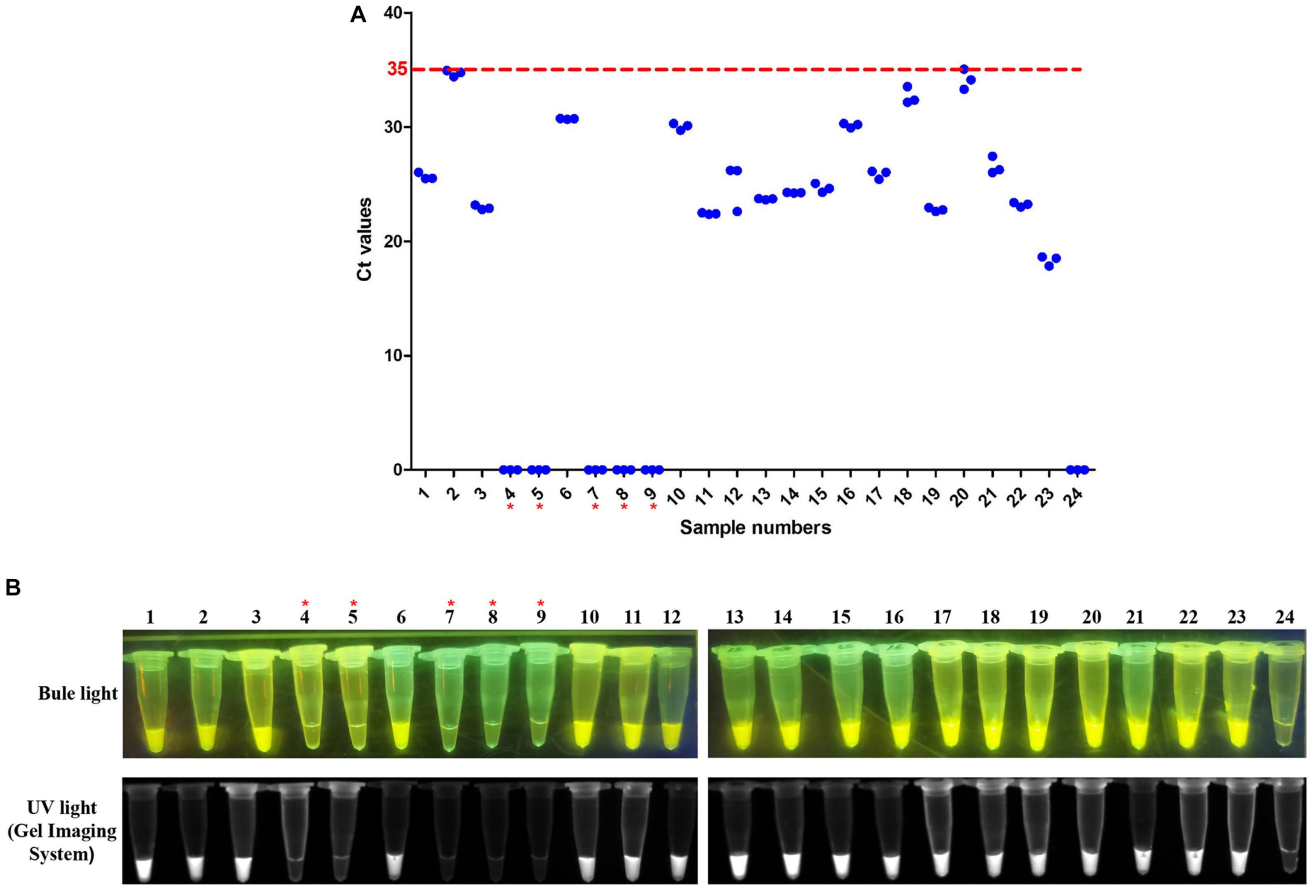
Evaluation of the RT-LAMP-based CRISPR-Cas12a assay on tissue samples. **(A)** SYBR Green qPCR assay on tissue samples. Numbers 1–22 represent tissue sample identification numbers. Numbers 23 and 24 represent positive samples and negative controls, respectively. A sample with an average Ct value ≤35 was positive, and a sample with an average Ct value >35 or undetectable DTMUV RNA level was negative. **(B)** RT-LAMP-based CRISPR-Cas12a assay on tissue samples. Lanes 1–22 represent tissue samples. Lanes 23 and 24 represent the positive sample and negative control, respectively. In each figure, the red asterisk indicates the negative samples identified by the two assays.

To further evaluate the RT-LAMP-based CRISPR-Cas12a assay, we tested 54 clinical samples (32 cloacal swabs and 22 tissue samples) taken from DTMUV-infected ducks. All clinical samples were first tested using SYBR Green qPCR previously established by our laboratory. Of these samples, 23 of 32 cloacal swabs (Sample numbers 2–4, 6–7, 9–10, 13–16, 18–26, 28–29, and 32) were positive for DTMUV infection by SYBR Green qPCR, but the other 9 cloacal swabs (Sample numbers 1, 5, 8, 11–12, 17, 27, 30, and 31) were negative (Figure 7A). Then, these samples were used to evaluate the RT–LAMP-based CRISPR-Cas12a assay. Our results showed that 23 of 32 cloacal swabs (Sample numbers 2–4, 6–7, 9–10, 13–16, 18–26, 28–29, and 32) were positive for DTMUV infection by the RT-LAMP-based CRISPR-Cas12a assay, but the remaining 9 cloacal swabs were negative (Figure 7B). Thus, the PPA and NPA of the RT-LAMP-based CRISPR-Cas12a assay compared to the SYBR Green qPCR were all 100% for DTMUV detection in 32 total cloacal swabs. Thereafter, we also tested 22 tissue samples by the two methods. As a result, 17 of 22 tissue samples (Sample numbers 1–3, 6, and 10–22) were positive for DTMUV infection by the RT-LAMP-based CRISPR-Cas12a assay and SYBR Green qPCR, but 5 of 22 tissue samples (Sample numbers 4–5 and 7–9) were negative, which indicated that there were 100%PPA and 100%NPA for detection of the 22 tissue samples using these two methods (Figures 8A,B). In conclusion, there was 100% concordance between the RT-LAMP-based CRISPR-Cas12a assay and SYBR Green qPCR for DTMUV detection in cloacal swabs and tissue samples.

## 4 Discussion

Molecular diagnostic methods, which rapidly detect DTMUV, facilitate the early detection of virus infection to avoid further outbreaks. Indeed, the PCR-based assays are more commonly used for virus detection among various diagnostic methods. Since the first outbreak of DTMUV in China in 2010, several PCR-based assays targeting the *E*, *NS3* and *NS5* genes have been developed to detect DTMUV, with limits of detection reaching 10 copies/μL, 1.82TCID_50_ and 0.001EID_50_/μL for viral RNA, respectively (Zang et al., 2012; Li, 2013; Wanzhe et al., 2016; Wang et al., 2017; Ninvilai et al., 2020). However, these PCR-based assays are not convenient for detecting a large number of clinical samples due to their complex steps and requirements for special equipment. Compared with them, the qPCR assays achieve high-throughput pathogen detection and provide a real-time readout via the qPCR instrument. Currently, some qPCR assays, such as one-step RT-qPCR assay targeting the 3′ terminal noncoding region (NCR), TaqMan-based qPCR assays targeting the *E* or *NS1* gene and SYBR Green qPCR assay targeting the *E* gene, have been exploited to detect DTMUV RNA or cDNA, and these assays have limits of detection from 10 to 2.67 × 10^2^ copies/μL (Yan L. P. et al., 2011; Yan et al., 2012; Yun et al., 2012; Wan et al., 2016; Chen G. Q. et al., 2018; Zhang et al., 2020; Li et al., 2023). Nonetheless, both PCR-based assays and qPCR assays take at least 2 to 4 h and require special equipment, which suggests that they are not convenient for the field detection of DTMUV. In addition, several RT-LAMP assays targeting the *E* or *NS5* gene were also established to detect DTMUV owing to their rapidity, visualization and convenience, and their limits of detection were from 10 to 4 × 10^2^ copies/μL for DTMUV RNA (Jiang et al., 2012; Wu et al., 2017; Wang et al., 2022). These assays are performed in a thermostatic water bath (61°C to 65°C), and their amplification can be completed within 50 min. However, the RT-LAMP assay easily causes false-positive results owning to cross-contamination and nonspecific amplification. Overall, these conventional detection methods, including PCR-based assays, qPCR assays and RT-LAMP assays, are difficult to widely use in clinical field detection due to their complex steps, requirements for special equipment or false-positive results.

In recent years, some studies have proven that CRISPR-Cas12a performs collateral cleavage on nontargeted ssDNAs when the Cas12a, crRNA and target DNA form a ternary complex (Li et al., 2018a). Under this condition, some isothermal amplification methods coupled with CRISPR-Cas12a had been established for pathogens detection (Chen J. S. et al., 2018; Li et al., 2018b; Tao et al., 2020; Hu et al., 2022). For example, an RPA-based CRISPR-Cas12a assay was developed for human papilloma virus (HPV) detection with a fluorescence readout, and its limited detection was 10 pM for HPV DNA (Chen J. S. et al., 2018). Within 1 h, this assay accurately identified the presence or absence of HPV in 25 patient samples. Meanwhile, another RT-LAMP-based CRISPR-Cas12a assay for hepatitis C virus (HCV) detection was also established, whose detectable concentration can reach 10 ng/μL for HCV RNA, and this assay can detect HCV within 1 h 30 min (Kham-Kjing et al., 2022). Therefore, these novel detection assays do not require special equipment and are more sensitive and specific than conventional PCR, qPCR and RT-LAMP assays. In this study, we developed an RT-LAMP-based CRISPR-Cas12a assay targeting the *C* gene for DTMUV detection. This assay shows several advantages over the existing methods based on conventional PCR, qPCR, and RT-LAMP. First, this assay only required some simple equipment, such as a water bath for isothermal amplification and CRISPR-Cas12a-mediated cleavage, and a blue or a UV light transilluminator for readout. Second, our RT-LAMP-based CRISPR-Cas12a assay has higher specificity than RT-LAMP solely because the Cas12a-crRNA complex specifically recognizes the target gene and discriminates nucleotide mismatches to avoid false-positive results (Chen J. S. et al., 2018). Third, our assay detects DTMUV RNA with a limited detection of 3 copies/μL, which is more sensitive than reported qPCR assays of DTMUV, including the SYBR Green qPCR assay (100 copies/μL) in our study (Yan L. P. et al., 2011; Yan et al., 2012; Yun et al., 2012; Wan et al., 2016; Chen G. Q. et al., 2018; Zhang et al., 2020; Li et al., 2023). Fourth, our assay can be completed within 2 h from RNA extraction to readout, which suggests that our assay is more suitable for rapid detection of DTMUV relative to conventional PCR and qPCR.

In addition, we tested 32 cloacal swabs and 22 tissue samples to further evaluate our RT-LAMP-based CRISPR-Cas12a assay. This assay showed 100% PPA and 100% NPA to SYBR Green qPCR for DTMUV detection in these clinical samples. Therefore, our assay can be applied for clinical field detection.

In summary, we successfully developed an RT-LAMP-based CRISPR-Cas12a assay to detect DTMUV RNA. This is the first report of DTMUV detection based on the combination of RT-LAMP with CRISPR-Cas12a. This novel method is suitable for clinical field detection owning to its visualization, high sensitivity and specificity.

## Data availability statement

The datasets presented in this study can be found in online repositories. The names of the repository/repositories and accession number(s) can be found in the article/Supplementary material.

## Ethics statement

The animal study was approved by the Laboratory Animal Ethics Committee of South China Agricultural University. The study was conducted in accordance with the local legislation and institutional requirements.

## Author contributions

YD: Data curation, Methodology, Software, Validation, Writing – original draft, Writing – review & editing, Investigation, Formal analysis. ZH: Writing – review & editing, Investigation. XL: Writing – review & editing, Investigation. MT: Writing – review & editing, Investigation. WL: Writing – review & editing, Investigation. SF: Writing – review & editing, Investigation. LZ: Writing – review & editing, Investigation. JZ: Writing – review & editing, Software. SY: Writing – review & editing, Investigation. FS: Writing – review & editing, Resources. PJ: Writing – original draft, Writing – review & editing, Conceptualization, Data curation, Formal analysis, Funding acquisition, Investigation, Methodology, Project administration, Resources, Supervision, Validation, Visualization.
