# Supplementary material for: Development of a reverse transcription loop-mediated isothermal amplification based clustered regularly interspaced short palindromic repeats Cas12a assay for duck Tembusu virus
Source: Front Microbiol. 2023 Nov 30;14:1301653. doi: 10.3389/fmicb.2023.1301653 (PMC10720249; doi:10.3389/fmicb.2023.1301653)
Supplement: Supplementary file 1 [file Data_Sheet_1.docx]

***Supplementary Material***

**Development of a reverse transcription loop-mediated isothermal amplification based** **CRISPR Cas12a assay for duck Tembusu virus**

**Yangbao Ding^1,2,3^, Zhanhong Huang^1^, Xinbo Li^1^, Mei Tang^1^, Weiqiang Li^1^, Siyu Feng^1^, Luxiang Zhao^1^, Junsheng Zhang^1^, Shichao Yuan^1^, Fen Shan^4*^, Peirong Jiao^1,2,3*^**

**Correspondence:**

Corresponding Authors: prjiao@scau.edu.cn; shanfen_gzzoo@sina.com.

**Supplementary Figure**


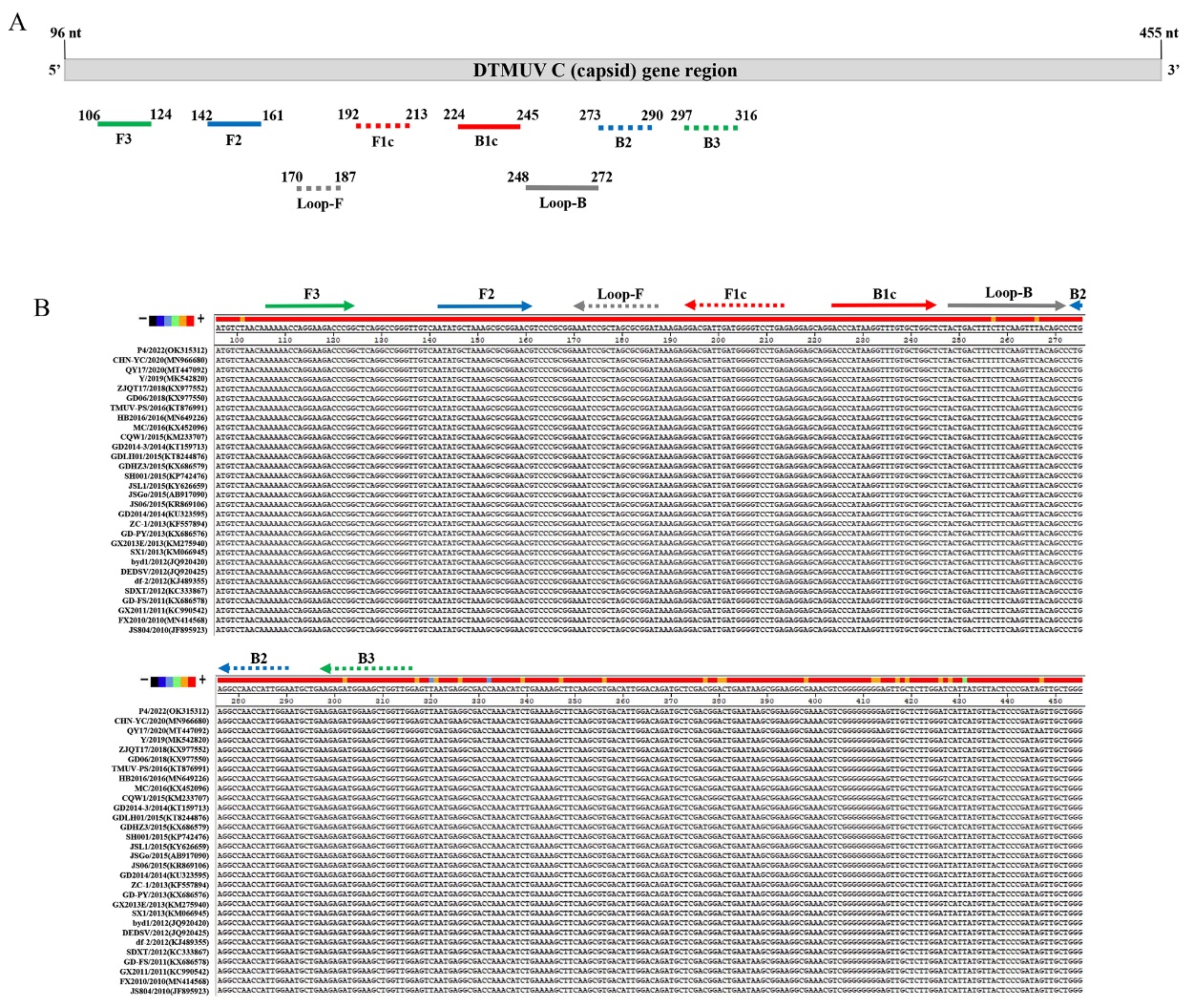


**Figure S1. Primers for the RT‒LAMP-based CRISPR‒Cas12a assay.** (A) RT‒LAMP primers indicated by different colors. The FIP primer is composed of F1c and F2, and the BIP primer is composed of B1c and B2. (B) Conservation analysis for *C* genes of thirty DTMUVs isolated in China. The sequences and locations of RT**‒**LAMP primers are shown on the *C* gene. Solid lines represent forward primers, and dotted lines represent reverse primers.
